# Genome Sequence of Acinetobacter towneri Strain DSM 16313, Previously Known as the Proposed Type Strain of Acinetobacter seohaensis

**DOI:** 10.1128/MRA.00690-21

**Published:** 2021-09-02

**Authors:** Shotaro Maehana, Hidero Kitasato, Masato Suzuki

**Affiliations:** a Department of Microbiology, School of Allied Health Sciences, Kitasato University, Kanagawa, Japan; b Department of Environmental Microbiology, Graduate School of Medical Sciences, Kitasato University, Kanagawa, Japan; c Regenerative Medicine and Cell Design Research Facility, School of Allied Health Sciences, Kitasato University, Kanagawa, Japan; d Antimicrobial Resistance Research Center, National Institute of Infectious Diseases, Tokyo, Japan; Montana State University

## Abstract

Acinetobacter species are widely distributed in the environment and clinical settings worldwide. We report the 2.99-Mbp draft genome sequence of Acinetobacter towneri strain DSM 16313, which was isolated from seawater in South Korea and proposed as a type strain of Acinetobacter seohaensis. Genome comparisons demonstrated that *A. seohaensis* should be reclassified as *A. towneri*.

## ANNOUNCEMENT

Acinetobacter towneri, belonging to the genus Acinetobacter, whose members are Gram-negative aerobic coccobacilli, is often isolated from water environments worldwide (1). This species has become increasingly important in recent years as a natural reservoir of antimicrobial resistance (AMR) genes (2–6). Of note, clinically relevant AMR genes, such as those for carbapenem-hydrolyzing enzymes (carbapenemases) and those for tigecycline-inactivating enzymes [*tet*(X)], have emerged in *A. towneri* via mobile gene elements, such as plasmids (2–6). Accumulation of AMR genes in environmental bacteria such as *A. towneri* and their transmission to human-pathogenic bacteria such as Acinetobacter baumannii pose a global public health threat. In this study, we performed genomic analysis of Acinetobacter seohaensis DSM 16313, a proposed type strain which was isolated from seawater from the Yellow Sea in South Korea in 2004. It was estimated to be similar to *A. towneri*, but the genome sequence of *A. seohaensis* was not publicly available (7).

Genomic DNA from strain DSM 16313 grown on tryptic soy agar was extracted using Genomic-tips and a genomic DNA buffer set (Qiagen). The Illumina sequencing library (paired-end format; insert size, 500 to 900 bp) was prepared using the Nextera XT DNA library prep kit (Illumina). Whole-genome sequencing was performed using the HiSeq X system (Illumina) with the HiSeq X Ten reagent kit v2.5 (300 cycles) (yield, 595.9 Mbp reads), followed by quality trimming of the Illumina reads using Trimmomatic v0.38.1 (https://github.com/usadellab/Trimmomatic) with default parameters (average quality required, 20). *De novo* assembly of the trimmed reads was performed using Shovill v1.1.0 (https://github.com/tseemann/shovill) with default parameters. The quality of the genome assembly was assessed using CheckM v1.1.3 (https://github.com/Ecogenomics/CheckM) with default parameters, and the genome completeness and contamination were estimated at 99.19% and 1.2%, respectively. The resulting draft genome sequence of DSM 16313 (GenBank accession no. BPEQ00000000) consisted of 298 contigs (average sequencing depth, 75×) with a total of 2,993,556 bp and a mean GC content of 41.3%. A total of 2,766 coding DNA sequences (CDSs) were annotated using the DFAST server (https://dfast.ddbj.nig.ac.jp/) with default parameters.

*In silico* DNA-DNA hybridization (DDH) analysis using the Genome-to-Genome Distance Calculator (GGDC) v2.1 (https://ggdc.dsmz.de/ggdc.php) with default parameters and average nucleotide identity (ANI) analysis using FastANI v1.3 (https://github.com/ParBLiSS/FastANI) with default parameters confirmed that *A. seohaensis* DSM 16313 (GenBank accession no. BPEQ00000000), *A. towneri* DSM 14962 (type strain; JHZH00000000), and *A. towneri* CIP 107472 (type strain in another bacterial bank; APPY00000000) are conspecific, with 78.4% DDH and 97.7% ANI (DSM 16313 compared with DSM 14962^T^) and 78.2% DDH and 97.6% ANI (DSM 16313 compared with CIP 107472^T^), respectively, according to their proposed minimal standards (≥70% DDH or ≥95% ANI) (8).

Thus, comparative genomic analysis in this study demonstrated that *A. seohaensis* should be reclassified as *A. towneri* and suggested that genome-level comparisons are essential for proposing novel bacterial species in phylogenetically diverse species, such as Acinetobacter.

### Data availability.

This whole-genome shotgun project has been deposited in DDBJ/ENA/GenBank under the accession no. BPEQ00000000. The version described in this paper is the first version, BPEQ01000000.1. The raw sequence data are available in the Sequence Read Archive under the accession no. DRX297109.
